# A milestone in the era of esports: The Olympics through the lens of virtual reality

**DOI:** 10.3389/fpsyg.2022.990189

**Published:** 2022-11-01

**Authors:** Bogdan Anðelić, Antonino Bianco, Nemanja Maksimović, Nikola Todorović, Patrik Drid

**Affiliations:** ^1^Sport and Exercise Sciences Research Unit, University of Palermo, Palermo, Italy; ^2^Faculty of Sport and Physical Education, University of Novi Sad, Novi Sad, Serbia

**Keywords:** esports, virtual reality, physical activity, video games, Olympics

## Introduction

Whether the International Olympic Committee (IOC) ever includes electronic sports (esports) in the official program of the Olympic Games, the popularity of competitive gaming continues to grow—sports present a modern audience powerhouse the world cannot fail to notice. By the end of 2025, the number of viewers in esports is expected to reach 640.8 million and revenue growth of 1,866.2 billion U.S. dollars (Gough, 2022). Over 170 colleges in the United States have established varsity esports teams and offer esports players' educational opportunities, including partial or full scholarships (Lyman, 2022). Esports has also found its place in schools, largely influencing the youth (Rothwell and Shaffer, 2019). Additionally, numerous NBA teams have entered the esports race, and even Usain Bolt owns an esports team (Gardner, 2022), perhaps better illustrating esports' global prevalence.

In 2021, the IOC took a step closer to the digital world, creating the Olympic Virtual Series (OVS). The main objective was to promote the development of virtual sports games and engage with the gaming communities worldwide. The OVS consisted of five different games: baseball (Powerful Pro Baseball 2020), cycling (Zwift), rowing (World rowing, using a rowing machine), sailing (Virtual Regatta), and motor racing (Gran Turismo). The IOC fused traditional elements of sport physicality and video games to uniquely incorporate physical activity (PA) to new audiences, connecting “e” and sports through the Olympic Movement (Palaar, 2021). Opposingly, the 2022 Asian Games (postponed to 2023 due to COVID-19) in Hangzhou will be the first official continental competition to include eight esports games played on computers, consoles, and even smartphone devices (Daniels, 2021).

While these actions by the IOC certainly took cognizance of esports and presented it to a world audience, none of the included games utilized virtual reality (VR) technology. Compared to the vast library of traditional esports games (e.g., Dota 2, League of Legends, Apex, CS:GO), VR games have a small selection of titles—which has affected the global VR prevalence in the gaming world. However, an excellent example is the VR game Onward, which (according to players) brought new features and a higher skill ceiling than traditional games, in addition to the physical embodiment and “immersiveness” for players (Turkay et al., 2021). VR is defined as an immersive and multisensory experience for the user, with the support of multimedia components such as VR headset and body tracking sensors (Gigante, 1993). As opposed to the 360° VR, which relies on the video recorded by the actual camera and then implemented into the system—this gives users a more realistic view and experience. However, both rely on the physical headset device with or without motion controllers. VR and the gaming industry joint became more evident as VR became a globally more available technology (e.g., Oculus Rift, HTC Vive, PlayStation) on the market. While the VR player base is not as big as Dota 2 or CS:GO, VR esports have a promising future with exponential growth, forecasted to reach 2.4 billion U.S dollars by 2024 (Clement, 2022). Besides games, VR technology has wide application in the rehabilitation of specific populations, psychology, psychiatry, education, and even sports performance (Lange et al., 2010; Salem and Elokda, 2014; Izard et al., 2018; Michalski et al., 2019; Park et al., 2019; Kaplan et al., 2021; Lee et al., 2021).

**Figure 1:**
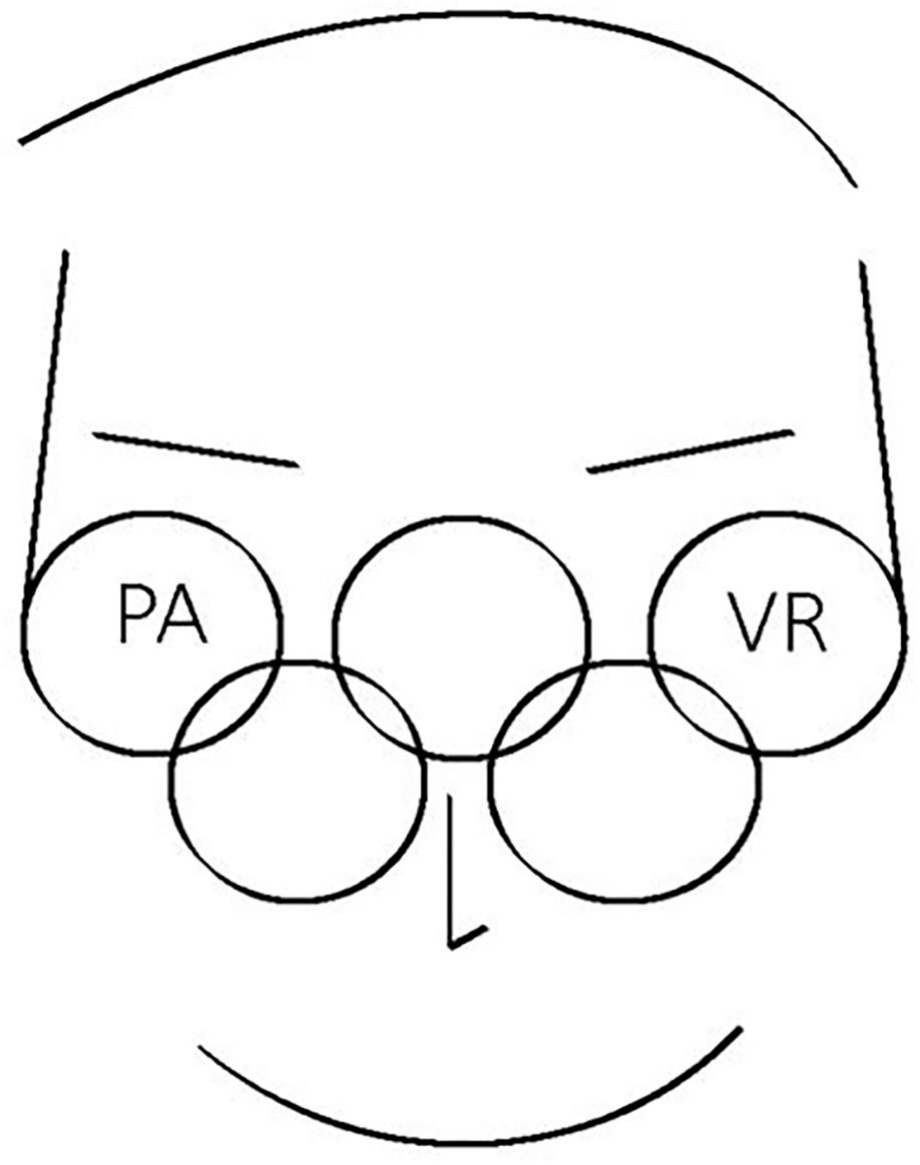
How do you perceive the world through the lens of virtual reality (VR)?

This paper aims to elicit ideas regarding the promotion of VR technology as a significant constituent of a milestone in the era of both physical and virtual sports, affording many new opportunities for the future of both esports and the Olympics (Figure 1).

## Discussion

To keep up with the modern era and accompanied hypokinesia, we need to develop on-par innovations: VR may be the right tool for promoting PA in the esports world (Sousa et al., 2022). For a moment, it is not hard to imagine first-person shooting (FPS) games like CS:GO (Rothery, 2021) or virtual sports games (e.g., Virtual Regatta or Gran Turismo) to run with the support of VR at the next OVS event in Singapore (Olympics, 2022). Exergames (EG) are an excellent example of a “healthier gaming” as they combine PA, and the use of modern technology in the gameplay. Depending on the game design, EG gameplay consists of PA and cognitive abilities supported by an immersive VR system in which players perform body movements to advance (Ketelhut et al., 2021). A similar approach has also been applied in VR esports—a mix of PA and esports enhanced by immersive VR technology (Martin-Niedecken and Schättin, 2020).

VR esports took a beaten path of traditional esports popularity and technological advancements. Nonetheless, VR esports have established several leagues, providing players opportunities to compete in different games. As VR technology advances and supports the wireless use, players can freely “roam” (e.g., location-based VR esports) and perform a variety of body movements (run, jump etc.) while playing (Turkay et al., 2021). For example, players can now experience a location based VR esport game (After-H Battle Arena) created by the Esports Virtual Arena (EVA). This competitive team-based FPS is played in a spacious (from 4,300 to 10,700 square feet) arena, allowing players to fully utilize wireless VR systems and move freely. Besides the FPS genre, multiplayer online battleground arena (MOBA) games like League of Legends or Dota2 could also benefit from VR systems (Ortiz and Reyes, 2020). Besides the FPS genre, players spend many hours practicing and mastering the game mechanics: it is a matter of time before esport coaches start implementing alternative training methods for players' development (Soler-Dominguez and Gonzalez, 2021)—both based on virtuality, novel VR-supported programs can be more engaging (Lakicevic et al., 2020). To draw a parallel, training specific sports such as basketball not only consists of training elements of the game but also includes several other developmental areas. Thus, the development of specific VR-supported games as a tool to enhance players' health and performance may be a more attractive approach for the esports community while promoting PA simultaneously—affecting future generations' perception of esports in the long run. Possibilities seem endless, as proper navigation of the technology and esports phenomenon has a huge potential effect on our society (Polman et al., 2018).

The road to the modern era with more focused VR esports may be the key to the puzzle: How to implement the layer of physical embodiment and multidimensional interactivity in esports. VR esports allows the players to enjoy the gaming experience and compete on the move—as opposed to sitting while playing non-VR esports (Dota 2 or LoL). This opinion aims to fill the gap between “e” and sports among the youth: promote VR in esports and VR esports to the IOC and vast gaming community, enrich the user experience—while repressing the dominant hypokinetic (seated) “gaming posture” in traditional esports. The OVS demonstrated the merge of advanced technology and PA in several games, showcasing esports from a different perspective: sailing, racing, and rowing simulators would potentially largely benefit from VR technologies and bring more enjoyment to the user (Faric et al., 2019). Game developers should be encouraged by high governing bodies and federations to advocate the safe and more efficient use of VR technology in the gaming community (Park and Lee, 2020).

Undoubtedly, the gaming industry is not going to disappear but may change its shape—affecting the global audience and especially the youth; research teams are obliged to conduct further work as technology does not look back (Polman et al., 2018). This paper should elicit awareness and invoke ideas of VR applicability in esports, and video games and potentially influence future (digital) events led by the IOC: if esports are on the road to the Olympics, could VR esports be a wildcard (Postma et al., 2022). Innovative and safe approaches are welcome and may largely mediate viewers, players, federations, research teams, and coaches to perceive the future of VR and esports, especially at imminent (e)sporting events such as the Olympics.

## Author contributions

Conceptualization and writing—original draft preparation: BA and AB. Writing—review and editing: BA, AB, NM, NT, and PD. Visualization: BA. Supervision and funding acquisition: PD. All authors have read and agreed to the published version of the manuscript. All authors contributed to the article and approved the submitted version.

## Funding

This work was supported by the Provincial Secretariat for Higher Education and Scientific Research (142-451-2594).

## Conflict of interest

The authors declare that the research was conducted in the absence of any commercial or financial relationships that could be construed as a potential conflict of interest.

## Publisher's note

All claims expressed in this article are solely those of the authors and do not necessarily represent those of their affiliated organizations, or those of the publisher, the editors and the reviewers. Any product that may be evaluated in this article, or claim that may be made by its manufacturer, is not guaranteed or endorsed by the publisher.
